# 2551. Assessing Meropenem Concentrations in Children with Septic Shock: Population-Based Pharmacokinetic (PK) Modeling of Meropenem Demonstrates Variability from Acute Kidney (AKI) to Augmented Renal Clearance (ARC)

**DOI:** 10.1093/ofid/ofad500.2168

**Published:** 2023-11-27

**Authors:** Jennifer Le, Julie Huynh, John S Bradley, Brandon Vo, Jeremiah Momper, Edmund Capparelli

**Affiliations:** University of California San Diego Skaggs School of Pharmacy and Pharmaceutical Sciences, San Diego, California; UCSD Scaggs School of Pharmacy and Pharmaceutical Sciences, San Diego, California; University of San Diego School of Medicine, Rady Children's Hospital, San Deigo, California; University of California, Riverside, Rosemead, California; UC San Diego, La Jolla, California; University of California San Diego, LA JOLLA, California

## Abstract

**Background:**

The primary objective of this study was to characterize meropenem (MERO) pharmacokinetics (PK) in critically ill infants and children with septic shock to develop dosing recommendations to achieve therapeutic drug exposure in this complex population with a spectrum of renal function from augmented renal clearance (ARC) to acute kidney injury (AKI).

**Methods:**

Children ≥4 weeks old hospitalized with septic shock requiring fluid resuscitation and pressors, receiving MERO 20 mg/kg IV every 8 hr as standard-of-care, were enrolled and studied prospectively from 2019 to 2023. Population PK modeling was used to derive Bayesian post-hoc PK parameters (NONMEM 7.3). Renal biomarkers (serum creatinine [SCr], cystatin C [CYS], serum neutrophil gelatinase-associated lipocalin [NGAL]) were evaluated as covariates. Estimated glomerular filtration rate (e-GFR) was calculated by the modified Schwartz equation.

**Results:**

A total of 304 MERO serum concentrations were included from 26 participants. Median age was 12.6 yr (range 1.2-19.6); weight 38.4 kg (10-98); SCr 0.36 mg/dL (0.09-2.57); CYS 448.9 mg/d (178.3-1824.1), and NGAL 176.05 ng/mL (29.3-1000). Median MERO serum concentration was 11 mcg/mL (range 0.033-217, interquartile range [IQR] 1.873-37.85). A two-compartment model with allometrically scaled weight on clearance (0.75) best described the data. Significant covariates (final model) were e-GFR, CYS, NGAL, age, diagnosis of appendicitis and albumin. Using the final model, the median CL was 0.15 (range 0.05-0.51, interquartile range [IQR] 0.06-0.25) L/hr/kg and V1 0.20 (0.07-0.57, IQR 0.12-0.31) L/kg, with GFR 139 (23-364, IQR 93-204) mL/min/1.73 m2 (Figure). The distribution of MERO concentrations, CL and GFR across quartiles documents wide variations in renal function observed in septic shock, from AKI to ARC (Figure).

Meropenem Serum Concentration, Meropenem Renal Clearance, and Glomerular Filtration Rate, by Quartiles

**Figure:**
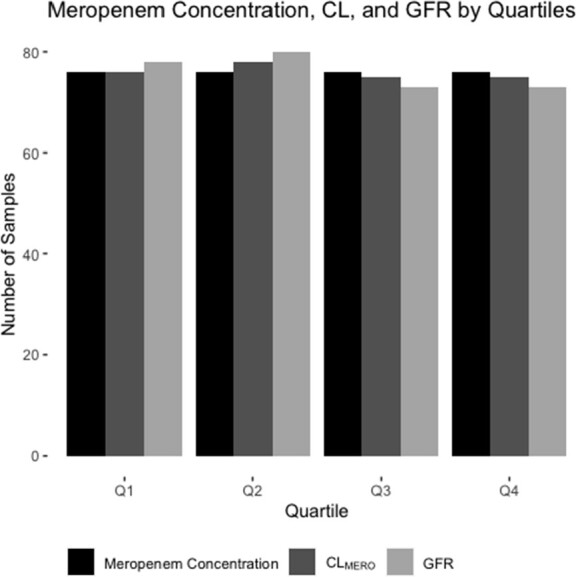

**Conclusion:**

Wide variation in MERO plasma exposure was documented in children with septic shock across the spectrum of renal function. GFR-based dosing recommendations may optimize MERO dosing and improve outcomes in this population.

This work was supported by NICHD grant 1 R01 HD095547-01

**Disclosures:**

**Edmund Capparelli, PharmD**, Melinta: Advisor/Consultant

